# Subjects with local allergic rhinitis can be identified by basophil activation test

**DOI:** 10.1186/2045-7022-3-S2-O18

**Published:** 2013-07-16

**Authors:** Paloma Campo, Carmen Rondon, Enrique Gomez, Natalia Blanca-Lopez, M Jose Torres, Esther Barrionuevo, Rocio Herrera, Luisa Galindo, Cristobalina Mayorga, Miguel Blancal

**Affiliations:** 1 Carlos Haya Hospital, Allergy Service, Malaga, Spain; 2 Carlos Haya Hospital, Allergy Research Laboratory, Malaga, Spain; 3 Infanta Leonor Hospital, Allergy Service, Madrid, Spain

## Background

Local allergic rhinitis (LAR) is characterized by negative skin testing and serum specific IgE. Diagnosis is based on nasal provocation test (NPT), very sensitive but time-consuming, and/or presence of local synthesis of specific IgE (sIgE) which shows low sensitivity (22%). The aim of the study was to evaluate the presence of specific D. pteronyssinus (DP) activation of basophils by basophil activation test (BAT) in subjects with confirmed LAR.

## Methods

BAT was performed in 43 subjects: 16 with confirmed LAR (positive NPT with DP, negative skin testing and sIgE to DP), 13 with allergic rhinitis (AR)(positive NPT with DP, positive skin testing and sIgE to DP) and 14 healthy controls (negative NPT, negative skin testing and sIgE to DP). To demonstrate a specific IgE mechanism of basophil activation, BAT with wortmannin pre-treatment was performed in four LAR patients.

## Results

BAT with DP was positive in 85% of AR patients and 50% of LAR subjects. BAT showed a substantial correlation with NPT in AR subjects (kappa index: 0.78, p=0.0001). The positive responses of the 4 LAR samples that underwent BAT with wortmannin became negative when this substance was added to the assay.

## Conclusions

BAT was able to detect 50% of LAR subjects to DP, being more sensitive than detection of nasal specific IgE. Responses were IgE-specific demonstrated by BAT-wortmannin assay. Further studies are needed to test this assay with other allergens.

